# Bronchoesophageal fistula diagnosed on modified barium swallow, a unique presentation

**DOI:** 10.1016/j.radcr.2022.09.057

**Published:** 2022-10-11

**Authors:** Mihir Karande, Matthew Kang, Frances Lazarow

**Affiliations:** Eastern Virginia Medical School, 825 Fairfax Ave, Norfolk, VA 23507, USA

**Keywords:** Contrast aspiration, Bronchoesophageal fistula, Barium swallow, Aspiration pneumonia, Contrast reflux

## Abstract

Modified barium swallow (MBS) studies, performed in conjunction with speech pathologists, are routinely performed to assess for aspiration. The narrow field of view over the area of interest limits assessment of pathology in the thoracic esophagus and airways. We report a case of a 79-year-old female with bronchoesophageal fistula diagnosed incidentally after abnormal findings on an MBS initially performed to assess for aspiration.

## Introduction

Bronchoesophageal fistula (BEF) can form as a sequelae of esophageal malignancy, commonly presenting with recurrent episodes of aspiration pneumonia. Lack of serosa around the esophagus can facilitate invasion of esophageal malignancy to adjacent structures within the mediastinum. BEF also occurs due to bronchogenic carcinoma, prolonged endotracheal intubation, and certain infections. The barium esophagram is considered the most sensitive in detecting BEF, but it may also be detected by CT imaging as well [1]. Recognition of BEF can have significant clinical importance as well, as the median survival after diagnosis of BEF is reported to be between 1 and 6 weeks [2].

## Patient presentation

A 79-year-old female resident of an assisted-living facility denying previous medical history presented to the hospital with shortness of breath associated with chest pain and mild hemoptysis for one week. The only medication she was taking was Naproxen for the pain. The patient had no relevant surgical history. On review of systems, the patient denied fever, epistaxis, palpitations, abdominal pain, or weight loss. The patient had a significant smoking history, but no history of alcohol use.

On physical examination, the patient appeared ill and was in acute respiratory distress. She had decreased breath sounds on the left side with crackles and wheezing along with tachycardia. The remainder of the physical exam was unremarkable.

**Fig 1:**
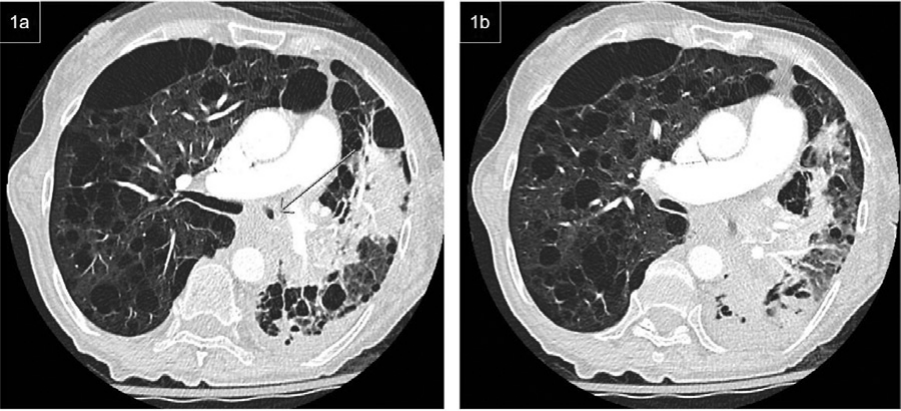
(A, B) Axial CT image shows infiltrate and postobstructive atelectasis in the left lower lobe with stenosis and debris in the left mainstem bronchus. Soft tissue attenuation around the mainstem bronchus raised suspicion for mass.

A CTA of the chest showed no evidence of a pulmonary embolism, but did reveal a large left lower lobe consolidation with a parapneumonic effusion. There was significant endoluminal debris and possible narrowing in the left mainstem bronchus with surrounding soft tissue that raised concern for a mass (Fig. 1).

On room air, the patient's O2 saturation was 80%, this was later managed with a non-rebreather mask at 15 L and later with a BiPAP, both of which provided minimal relief. The patient was later transferred to the ICU, where she received O2 through a high flow nasal cannula and was given Piperacillin/Tazobactam and Linezolid for pneumonia. Subsequent chest radiographs revealed advanced emphysema, leftward mediastinal shift due to atelectasis, and worsening right basilar opacities.

**Fig. 2:**
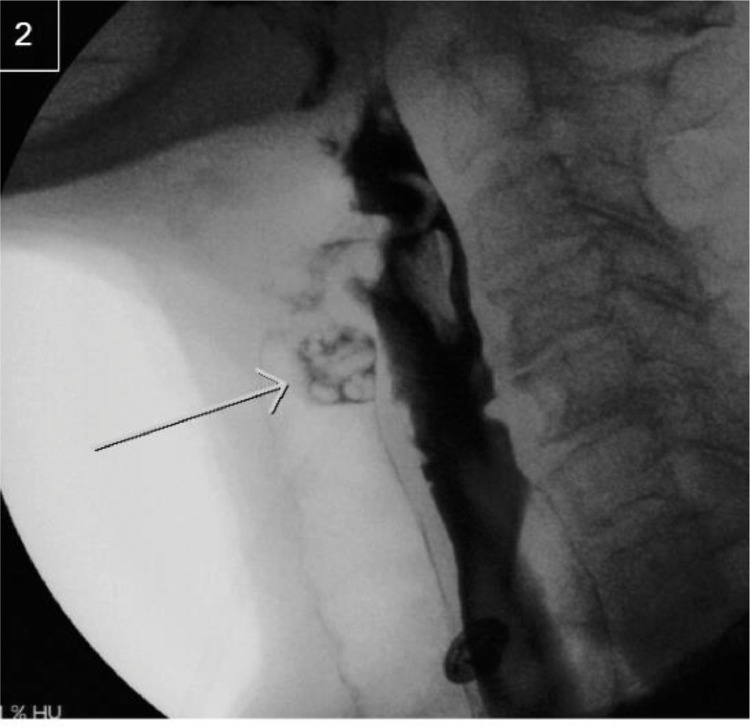
A small amount of aspirate with thin consistency barium swallow.

**Fig. 3:**
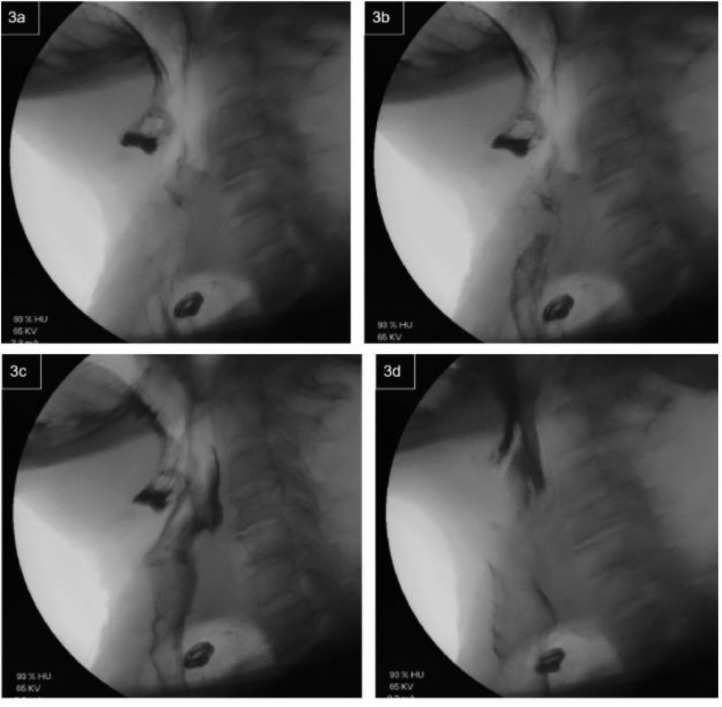
(A-D) The following images demonstrate a relatively large amount of barium moving retrograde through the trachea during cough with subsequent emesis. Given the disparity between amount of contrast aspirated versus the amount moving retrograde through the trachea, further inspection with a full esophagram was performed.

Given concerns of aspiration, a modified barium swallow study was performed in conjunction with speech pathology. The patient had no aspiration with pudding consistency barium. She had a small amount of aspiration with thin consistency barium (Fig. 2) and then began coughing. Contrast material was seen moving retrograde through the trachea and into the pharynx, a much larger volume than was aspirated (Fig. 3). The presence of this large amount of contrast within the trachea led to concerns of fistulous communication between the enteric and pulmonary tracts.

**Fig. 4:**
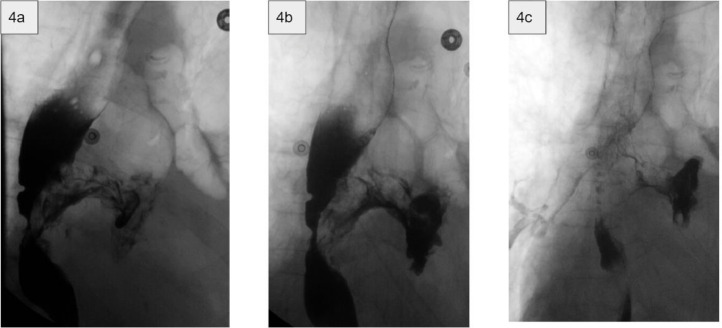
(A-C) Imaging shows a left sided concentric filling defect from extrinsic compression causing pronounced stenosis of the mid-distal esophagus. Multiple smaller filling defects are also noted in this region. Swallowed contrast is noted outside the lumen of the esophagus with static appearance in a fluid collection over the left hilum. Additional images after the coughing episode shows barium contrast outlining the trachea and right mainstem bronchus. Given these findings, a paraesophageal mass with the development of a bronchoesophageal fistula was diagnosed. The mass presumably invaded the esophagus near this level circumferentially, causing the left and right sided impressions upon the esophagus.

A subsequent esophagram was performed (Fig. 4). This demonstrated severe luminal narrowing of the esophagus and a large concentric filling defect at the T8-T10 level. Extraluminal contrast was noted to the left of the esophagus extending as a narrow band of contrast into a collection in the left mediastinum. After a small delay and additional coughing fit, a small layer of contrast was noted to outline the trachea and right mainstem bronchus. A focal area of stenosis of the proximal left mainstem bronchus was also seen.

Based on these findings, a BEF and paraesophageal mass within the lower posterior mediastinum were diagnosed. Given the location and patient history, an invasive distal esophageal malignancy or pulmonary malignancy was suspected. Endoscopic evaluation with biopsy was suggested; however, these were not pursued due to her limited prognosis and extreme debility along with the patient's wishes not to pursue any further aggressive therapies or surgeries. The patient continued with supportive care and chose to seek hospice care due to her terminal diagnosis.

## Discussion

Bronchoesophageal fistula (BEF) is a rare sequelae of esophageal cancer, seen in 5%-15% of patients with esophageal cancer [1]. BEF is also observed in 1% of patients with bronchogenic carcinoma. It is seen less commonly in patients with prolonged endotracheal intubation, endoscopic interventions, and infections, from tuberculosis, HIV, and syphilis [3].

Most commonly, BEF presents with dysphagia, recurrent pulmonary infections, chest pain, hemoptysis, malnutrition, coughing after oral fluid intake (Ono's sign), and sepsis [2]. Increased mortality from BEF is the result of recurrent aspiration pneumonia. Recurrent aspiration and paroxysmal coughing can contribute to decreased oral intake as well, leading to malnutrition [4]. Increased mortality from BEF is most attributable to recurrent aspiration pneumonia [2]. In patients receiving positive pressure ventilation, gas forced through the fistula leads to gastric dilation and splinting of the diaphragm. In terms of imaging, the barium esophagram is considered the most sensitive exam for detection of BEF, providing a correct diagnosis 78% of the time [1]. Definitive imaging findings of BEF can be subtle, as demonstrated by this case presentation. A similar process of BEF discovery was documented by Han et al. who noted a large volume of reflux through the trachea without any documented aspiration [5].

Esophageal carcinoma typically appears as an intramural mass or localized wall thickening on CT imaging. On visualization of the cancer, it can exist as a circumferential paraesophageal mass and it may or may not have intraluminal narrowing. The spread of the esophageal carcinoma occurs through lymphatics or hematogenously. The lymphatic drainage occurs through 2, bidirectional lymphatic plexuses, one in the mucosa and one in the muscular layer. Because of this, lymphatic fluid from any portion of the esophagus may spread to any regional lymph nodes. A distal mass is just as likely to spread to the bronchial lymph nodes as a proximal mass. Esophageal carcinoma spreads hematogenously to the liver, lungs, bones, adrenal glands, kidneys, and brain [6]. As the esophagus lacks a serosa, no anatomic barrier exists between mucosa and mediastinum, allowing early local invasion. Abnormal soft tissue appearance along the trachea or bronchus increases suspicion for invasion [7].

Signs of communication between the respiratory tract and esophagus can be subtle or suboptimally demonstrated by CT. This case follows an atypical presentation of BEF during a routine MBS initially performed for aspiration pneumonia. The finding of interest was outside the field of view initially and retrograde contrast within the trachea could have been dismissed as previously aspirated contrast. This case highlights the importance of the radiologist maintaining a high degree of awareness and critical thinking while performing a MBS, a relatively routine study with limited potential for identifying pathology originating from an intrathoracic source.

## Patient consent

Appropriate permissions to include case details and images were received prior to conducting the case report.
